# Inhibition of the JAK/STAT Signaling Pathway in Regulatory T Cells Reveals a Very Dynamic Regulation of Foxp3 Expression

**DOI:** 10.1371/journal.pone.0153682

**Published:** 2016-04-14

**Authors:** Jérémie D. Goldstein, Aude Burlion, Bruno Zaragoza, Kélhia Sendeyo, Julia K. Polansky, Jochen Huehn, Eliane Piaggio, Benoit L. Salomon, Gilles Marodon

**Affiliations:** 1 Sorbonne Universités, UPMC Univ Paris 06, UMR-S CR7, Centre d’Immunologie et des Maladies Infectieuses (CIMI), INSERM U1135, CNRS ERL 8255, Paris, France; 2 Department of Experimental Immunology, Helmholtz Centre for Infection Research, Braunschweig, Germany; 3 INSERM U932, Institut Curie, Paris, France; Jackson Laboratory, UNITED STATES

## Abstract

The IL-2/JAK3/STAT-5 signaling pathway is involved on the initiation and maintenance of the transcription factor Foxp3 in regulatory T cells (Treg) and has been associated with demethylation of the intronic Conserved Non Coding Sequence-2 (CNS2). However, the role of the JAK/STAT pathway in controlling Foxp3 in the short term has been poorly investigated. Using two different JAK/STAT pharmacological inhibitors, we observed a detectable loss of Foxp3 after 10 min. of treatment that affected 70% of the cells after one hour. Using cycloheximide, a general inhibitor of mRNA translation, we determined that Foxp3, but not CD25, has a high turnover in IL-2 stimulated Treg. This reduction was correlated with a rapid reduction of *Foxp3* mRNA. This loss of Foxp3 was associated with a loss in STAT-5 binding to the CNS2, which however remains demethylated. Consequently, Foxp3 expression returns to normal level upon restoration of basal JAK/STAT signaling *in vivo*. Reduced expression of several genes defining Treg identity was also observed upon treatment. Thus, our results demonstrate that Foxp3 has a rapid turn over in Treg partly controlled at the transcriptional level by the JAK/STAT pathway.

## Introduction

CD4^+^CD25^+^Foxp3^+^ regulatory T cells (Treg) are essential actors of the immune system [1]. Expression of the transcription factor Foxp3 is a prerequisite for their suppressive activity, since patients carrying mutations altering the expression or function of Foxp3 may develop IPEX (Immunodysregulation polyendocrinopathy enteropathy X-linked syndrome, a severe autoimmune disease [2]. Also, *Scurfy* mice, presenting a mutation in the *foxp3* gene develop an IPEX-like disease [3,4]. Because Foxp3 is essential for function, proliferative potential and metabolic fitness of Treg, it is essential to gather more information on its regulation at the transcriptional and post-transcriptional levels.

Genetically engineered mice have been instrumental in deciphering the molecular pathways leading to Foxp3 expression. Mice deficient in various members of the IL-2/CD122/JAK3/STAT-5 signaling pathway present a profound decrease in thymic and peripheral Treg [5–7]. These results have been integrated into a model where IL-2 would represent the main driver for Foxp3 transcription in the thymus and the periphery [8]. IL-2 may affect Foxp3 regulation through binding of the transcription factor STAT-5 to the *foxp3* promoter and to the Treg-Specific Demethylated Region (TSDR) [6,9,10] an enhancer of the *Foxp3* gene that is specifically demethylated in Treg [11]. This TSDR region (also known as Conserved Noncoding Sequence-2 (CNS2) [12]) is required for the maintenance of Foxp3 protein expression and stability of the Treg lineage, but not the initiation of Foxp3 mRNA transcription [12–14]. Furthermore, Foxp3^+^ cells can be generated in the thymus without IL-2 but failed to maintain in the periphery [15,16], leading to the hypothesis that IL-2 might be more important for Treg survival in the periphery than for initiating Foxp3 expression in the thymus. Adding to this complexity is the emerging view that Treg is a plastic lineage, able to convert to Teff in certain conditions. For instance, Treg injected in lymphopenic mice converts to Foxp3^-^ cells few weeks after and IL-2 is able to prevent this conversion [17]. Since then, numerous examples of Treg conversion to effector cells in inflamed tissues have been shown [1]. This conversion may depend on limited IL-2 availability in the inflamed tissues [18,19]. Indeed, the role of an optimal IL-2 signal to preserve CNS2 'activity' via recruitment of STAT-5 in dividing Treg has been clearly demonstrated [13,14]. Also, the role of IL-2 in preventing Treg conversion in vivo has been shown [20]. However, the effect of CNS2 deletion on Foxp3 stability was reported weeks after *in vivo* transfer of modified cells and days after their *in vitro* activation although fine tuning of the immune response would require a much more rapid adaptation to the inflammatory milieu. Thus, the impact of IL-2 signaling on short-term regulation of Foxp3 and how it relates to the status of CNS2 methylation in primary Treg is unknown.

Here, we used pharmacological inhibitors to block the JAK/STAT pathway in highly purified Treg from normal mice activated by IL-2 *ex vivo*. This strategy allows to determine the impact of the JAK/STAT pathway on Foxp3 expression and CNS methylation on a short time scale and in a controlled manner using cells from normal mice. Our results reveal a surprisingly high turnover of Foxp3, regulated at the mRNA level and identify a new subset of Treg with low expression of Foxp3 but with a demethylated CNS2, that may represent precursors of 'exTreg'.

## Results

### Blockade of JAK3/STAT-5 signaling pathway leads to a rapid down modulation of Foxp3 in Treg

**Fig 1:**
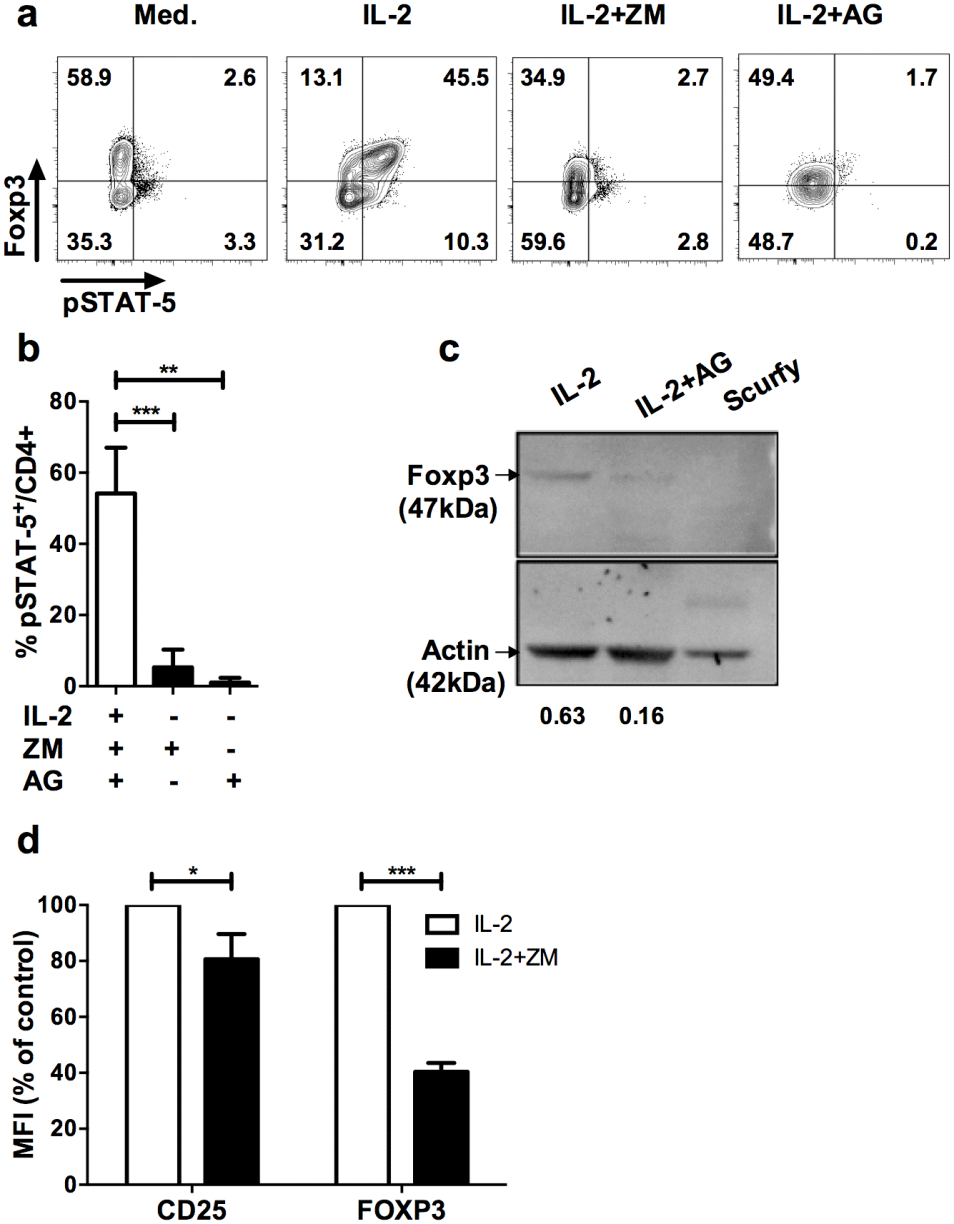
Blockade of JAK/STAT signaling pathway leads to down modulation of Foxp3 in Treg. (**a**) CD25-enriched T cells were cultured for one hour in complete medium alone (Med.), with IL-2 (IL-2), or IL-2 supplemented with ZM-39923 (IL-2+ZM) or AG-490 (IL-2+AG). Profiles shown are gated in CD4^+^ cells and are representative of 4 independent experiments. (**b**) Frequencies of pSTAT5^+^ cells among CD4^+^ cells cultured for one hour with IL-2 alone (IL-2) or in presence of IL-2 and the indicated JAK inhibitors (ZM, AG). Results are compiled from 4 independent experiments. **(c)** In vitro expanded CD4^+^GFP^+^ Treg were treated with IL-2 and with either 100ug/mL of AG490 (IL-2+AG) or vehicle control (absolute ethanol; EtOH) for 2 hrs (IL-2). Proteins were extracted in lysis buffer and blotted followed by anti-Foxp3 and anti-actin staining. Intensity values were normalized to the actin band in each blot. The specificity of the Foxp3 staining is attested by the absence of the Foxp3 band in extracts from splenocytes of a scurfy (genetically deficient for Foxp3) mouse. **(d)** Human CD4^+^CD25^+^ cells were enriched from PBMC of healthy donors by magnetic sorting and treated with IL-2 (600 IU/mL) with or without ZM (50 μM) for 1h. MFI of CD25 and FOXP3 in human CD4^+^CD3^+^ cells after treatment with IL-2 alone (IL-2) or IL-2 in presence of ZM (IL-2+ZM). The MFI of Foxp3 and CD25 shown were normalized by the MFI of control cultures. These data are compiled from 3 independent experiments. Statistical significance was tested using Student t-test (***p<0.001, **p<0.01, *p<0.05).

To determine the physiological role of the JAK3/STAT-5 signaling pathway in established Treg, we performed *in vitro* experiments in which we blocked IL-2-induced phosphorylation of STAT-5 in purified Treg with specific JAK3 inhibitors. We performed our study with two inhibitors of the JAK3/STAT-5 signaling pathway, ZM39923 (ZM) or Tyrphostin/AG490 (AG). ZM has been described as the most specific JAK3 inhibitor whereas AG targets JAK2 and JAK3 [21]. As we previously reported [22], IL-2 induced preferential phosphorylation of STAT-5 in Foxp3^+^ cells compared to Foxp3^-^CD4^+^ T cells in enriched Treg (Fig 1a). As expected, ZM and AG inhibitors completely prevented pSTAT5 induction by IL-2 (Fig 1b). We noticed that the proportion of Foxp3^+^ cells also decreased following one-hour treatment, apparently due to the down modulation of Foxp3 expression (Fig 1a). Indeed, we observed that the Foxp3 protein was reduced 4-fold upon treatment of highly pure Treg sorted from Foxp3-GFP reporter mice [23] compared to ethanol vehicle control (Fig 1c), suggesting that JAK inhibitors led to a rapid loss of Foxp3 in Treg. Importantly, reduction in Foxp3 expression upon JAK3 inhibition was also observed in purified human CD25^+^ cells (Fig 1d), showing that the effect was not restricted to murine Treg. Because we observed a similar loss of Foxp3 using murine and human Treg with both inhibitors, AG and ZM were used indifferently for the rest of the work.

### Reduced Foxp3 by JAK inhibitors is independent of cell death

**Fig 2:**
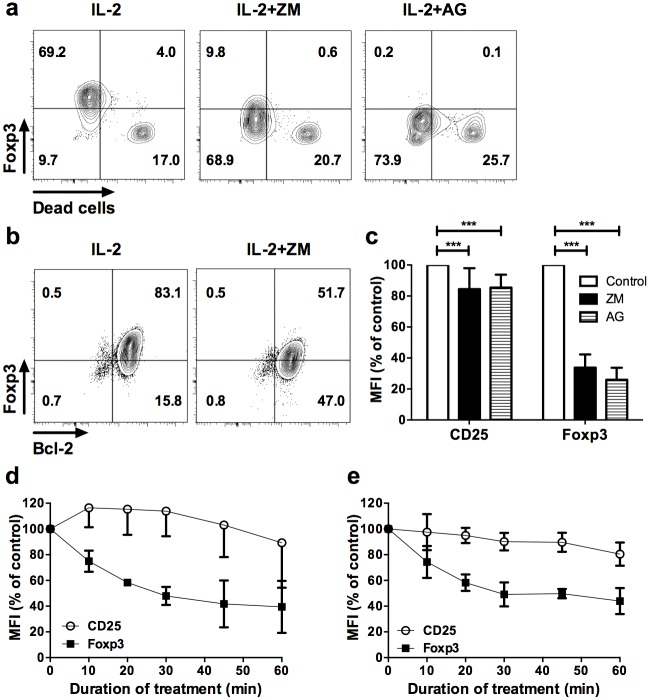
Reduced Foxp3 by JAK/STAT inhibitors is independent of cell death. **(a)** CD4^+^GFP^+^ T cells sorted from Foxp3-GFP transgenic mice were stimulated with anti-CD3/CD28 beads for 8 days and treated with vehicle and IL-2 (IL-2), or IL-2 supplemented with ZM (IL-2+ZM) or AG (IL-2+AG) JAK inhibitors for two hours. Cells were then stained with a dye allowing exclusion of dead cells by flow cytometry compatible with Foxp3 intracellular staining. Profiles are representative of 6 and 7 independent experiments for AG and ZM inhibitors, respectively. **(b)** Foxp3 and Bcl-2 expression in gated CD4^+^CD25^+^ cells from expanded CD4^+^GFP^+^ cells after 2 hour treatment with vehicle control and IL-2 (IL-2) or ZM in presence of IL-2 (IL-2+ZM). Similar results were observed in 3 independent experiments. **(c)** Median fluorescence intensity (MFI) of CD25 and Foxp3 in dead cell–excluded subset, represented as percentages of the MFI relative to Foxp3 staining in vehicle control condition (control) after two hours treatment with the indicated inhibitors. The results are compiled from 6 (AG) and 7 (ZM) independent experiments. **(d)** Freshly isolated or **(e)** anti-CD3/CD28 activated CD4^+^GFP^+^ cells from Foxp3-eGFP reporter mice were treated for the indicated times with either ZM or AG inhibitors. MFI of CD25 and Foxp3 are relative to the MFI of vehicle control conditions. These data are compiled from 3 independent experiments. Statistical significance was tested using Student t-test (***p<0.001, **p<0.01, *p<0.05).

One possibility to explain reduced Foxp3 in Treg would be that Foxp3^+^ cells preferentially died upon treatment. In order to investigate this hypothesis, we treated sorted Foxp3^+^ cells with JAK inhibitors and used a cell viability dye compatible with Foxp3 staining. Importantly, loss of Foxp3 expression upon ZM or AG treatments occurred within live cells (Fig 2a). In addition, decreased Foxp3 expression upon treatment with ZM did not correlate with alteration of the anti-apoptotic factor Bcl-2 (Fig 2b), in contrast to dead cells that invariably lost Bcl-2 expression (not depicted). Thus, after exclusion of dead cells from the analysis, we observed a 60 to 70% decrease of the Foxp3 Median of Fluorescence Intensity (MFI) compared to untreated controls in live CD4^+^ cells after one hour of treatment. Compared to Foxp3, CD25 expression was less affected by the treatments (Fig 2c). Thus, loss of Foxp3 expression upon JAK3 blockade was independent of cell death after one hour of treatment.

To investigate the regulation of Foxp3 by the JAK/STAT pathway on a shorter time scale, we monitored the reduction of Foxp3 expression with the AG inhibitor over a period of one hour at 5 different time points. We observed a reduction in the MFI of Foxp3 in freshly isolated Treg as early as 10 min of treatment, which became more pronounced overtime. In contrast, CD25 was only marginally affected by the treatment over this time period (Fig 2d). The same profiles were observed if the treatment was applied to CD3/CD28-activated Treg (Fig 2e), showing that the TCR and/or co-stimulatory signaling were unable to prevent the rapid reduction of Foxp3 upon JAK/STAT signaling inhibition. Of note is that a similar reduction in Foxp3 was observed in TGF-ß-induced Treg *in vitro* and that addition of TGF-β did not prevent the loss of Foxp3 in Foxp3-GFP^+^ Treg (S1 Fig). These results show that neither TCR nor TGF-ß signaling were able to prevent the action of JAK/STAT inhibitors.

### Rapid turn over of Foxp3 protein and mRNA in Treg

**Fig 3:**
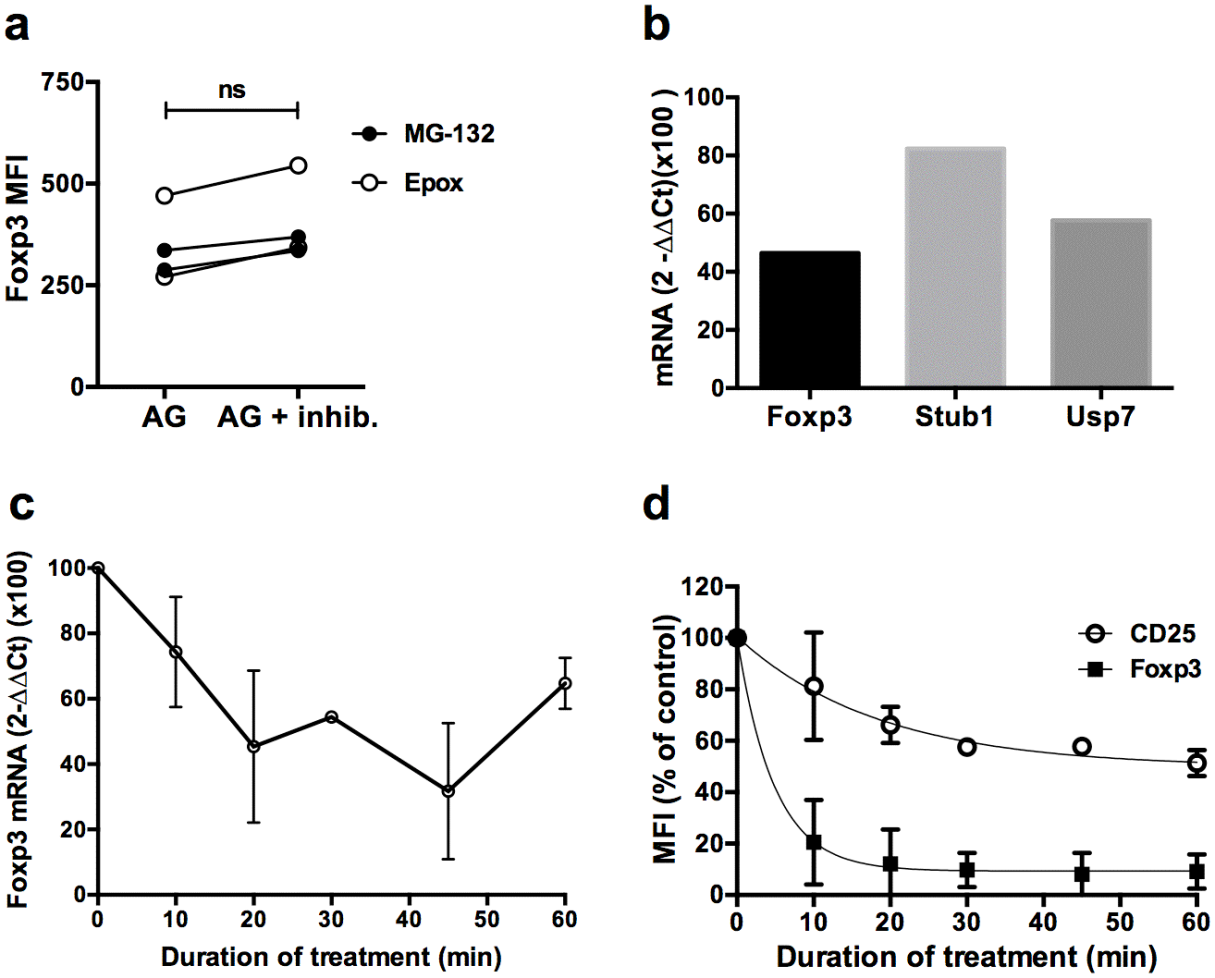
Rapid turn over of the Foxp3 protein and mRNA in Treg. **(a)** MFI of Foxp3 on GFP^+^ Treg treated with proteasome inhibitors MG-132 or Epoxomycine (Epox) in presence of IL-2 and AG for 2 hrs. Cells were pretreated during 2 hrs with the proteasome inhibitors before adding AG. **(b)** Foxp3, USP7 and STUB1 mRNA were quantified using the TaqMan RT-PCR assay normalized to vehicle control conditions 45 minutes after AG-treatment in expanded CD4^+^GFP^+^ cells by the 2^-ΔΔCt^ method (see method section). This result originate from a single experiment performed in triplicates. **(c)** Foxp3 mRNA was assessed by quantitative RT-PCR normalized to vehicle control conditions at the indicated times after AG-treatment in expanded CD4^+^GFP^+^ cells by the 2^-ΔΔCt^ method (see method section). The experiment was repeated 3 times. Each dot represent the mean ± SDEV of results obtained in 3 independent experiments. Two outliers were removed from the calculation **(d)** MFI of Foxp3 and CD25 in cycloheximide-treated sorted activated CD4^+^GFP^+^ cells relative to the vehicle control at the indicated times. Results are compiled from 3 independent experiments.

Decrease in Foxp3 at the protein level could be due to reduced input, i.e reduced mRNA transcription, and/or increased output, i.e increased degradation of the protein. To investigate these possibilities, we tried to prevent loss of Foxp3 induced by the AG-490 JAK inhibitor with two different proteasome inhibitors. However, MG-132 or Epoxomycine had only a moderate effect at preventing Foxp3 reduction upon AG treatment (Fig 3a), indicating that most of the reduction of Foxp3 by AG was not due to increased proteasomal degradation. Stability of Foxp3 has been linked to the activity of the deubiquitinase USP7, protecting Foxp3 from the proteasome [24]. We thus explored whether JAK/STAT inhibition would also impact expression of USP7. A quantitative RT-PCR analysis of purified Treg 45 min after treatment with AG revealed a significant loss of USP7, to the same extent than Foxp3 (Fig 3b). Interestingly, the STUB1 ubiquitinase mRNA, described as an inducer of Foxp3 proteasomal degradation [25], was not changed after treatment. Overall, these results indicate that proteasomal degradation of Foxp3 upon JAK/STAT inhibition might operate through diminished activity of the Foxp3-protecting USP7 deubiquitinase but that was not responsible for most of the changes.

We thus performed quantitative RT-PCR to assess whether loss of Foxp3 would be better explained by reduced transcription of Foxp3 mRNA. We observed a very similar reduction kinetics for Foxp3 mRNA than for the protein by TaqMan qPCR. Foxp3 mRNA was reduced by half in the first 20 min after treatment compared to vehicle-treated cells (Fig 3c). Thus, JAK/STAT signaling inhibition rapidly impacted Foxp3 mRNA transcription and /or stability, which in turns translated to reduced Foxp3 protein levels. To determine whether this rapid loss of the protein was reproduced in the absence of mRNA translation, we treated Foxp3^+^GFP^+^ Treg with a subtoxic dose of cycloheximide (CHX), an mRNA translation inhibitor. We observed a sharp decrease in Foxp3 expression as early as 10 min of treatment and an almost complete disappearance of the staining in 30 minutes with that dose. The decrease in the MFI of CD25 was much slower and less pronounced than Foxp3 (Fig 3d). Thus, this experiment confirms that the Foxp3 protein has a rapid turnover in Treg, that depends of continuous mRNA translation.

### Reduced Foxp3 in Treg by JAK inhibitors is not due to remethylation of the CNS2 and is reversible

**Fig 4:**
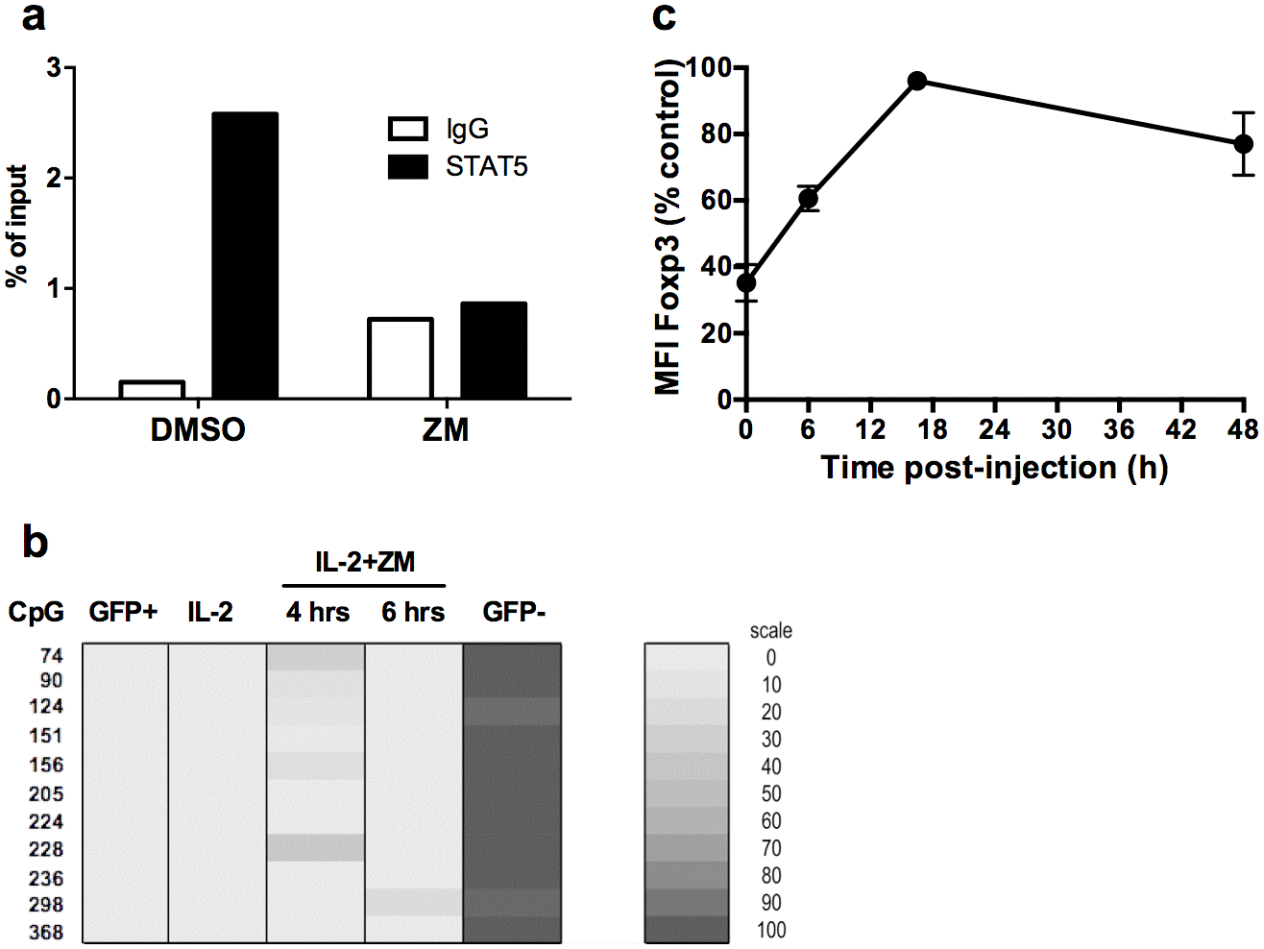
Loss of Foxp3 is not due to remethylation of the CNS2 and is reversible. **(a)** STAT5 binding on a putative binding site in the TSDR enhancer of the *foxp3* gene was assessed by chromatin immunoprecipitation with control IgG or anti-STAT-5 antibodies. Quantification of binding was performed as described in Methods section. These data are from one representative experiment out of two. (**b**) Methylation status of the *Foxp3* TSDR was determined by bisulphite sequencing. Expanded CD4^+^GFP^+^ T cells were treated with IL-2 and ZM (IL-2+ZM) for 4 (4 hrs) or 6 (6 hrs) hours. Freshly purified CD4^+^GFP^+^ (GFP^+^), CD4^+^GFP^-^ (GFP^-^), or untreated expanded cells (IL-2) were used as controls. These data are representative of two independent experiments. Each line indicate the position in the Foxp3 locus (CpG), according to Floess et al. [11]. A shaded scale of grey illustrates the frequency of each methylated CpG. **(C)** For *in vivo* analysis, expanded CD4^+^GFP^+^ cells treated or not with AG for two hours were injected into congenic C57BL/6 mice. Animals were euthanized at the indicated times after transfer. The MFI of Foxp3 was normalized to vehicle-treated control cells.

Since STAT-5 is a major player of Foxp3 transcription by binding to multiple regulatory regions in the *Foxp3* gene, release of STAT-5 from the Foxp3 gene was one possible explanation for reduced mRNA transcription. Because the CNS2 is essential for Foxp3 stability, we determined whether STAT-5 was released from a putative binding site within the CNS2 (STAT1-binding site region 418–426 according to Floess et al. [11] or the GAS (IFN-**G**amma **A**ctivated **S**ites) motif 4384–4393 according to Kim and Leonard [26]. We could demonstrate that STAT-5 was indeed bound to the CNS2 at this location in vehicle-treated Treg cells. This specific binding was lost following JAK/STAT signaling inhibition (Fig 4a), showing that STAT-5 was released from the CNS2.

Since the CNS2 is fully demethylated *ex vivo* in isolated Treg and methylated in conventional T cells [11], we sought to determine whether reduced Foxp3 expression was associated with remethylation of the CNS2. As expected, we observed a fully demethylated CNS2 in freshly purified CD4^+^GFP^+^ cells and a fully methylated TSDR in CD4^+^GFP^-^ cells (Fig 4b). The methylation status of the CNS2 was not significantly affected 4 or 6 hours after treatment with ZM relative to untreated cells (Fig 4b). At this time, Foxp3 expression was still low in viable Treg (not shown). Thus, the loss of Foxp3 did not originate from increased methylation of the CNS2, suggesting that the rapid loss of Foxp3 described above was also unlikely to be due to remethylation of the CNS2.

The lack of CNS2 remethylation upon JAK/STAT inhibition suggests that the loss of Foxp3 could be reversible upon restoration of this signaling pathway. To determine whether loss of Foxp3 was reversible, AG-treated Treg cells having lost 70% of Foxp3 were transferred into normal congenic mice to restore the basal JAK/STAT signaling that Treg may encounter *in vivo*. A sharp increase in Foxp3 levels was observed 6 hours after transfer, and the MFI of Foxp3 was indistinguishable from untreated cells overnight after transfer *in vivo* (Fig 4c). Thus, the loss of Foxp3 expression upon transient inhibition of IL-2 signaling was reversible upon restoration of basal JAK/STAT signaling *in vivo*.

### Global impact of JAK/STAT signaling inhibition in Treg at the molecular level

**Fig 5:**
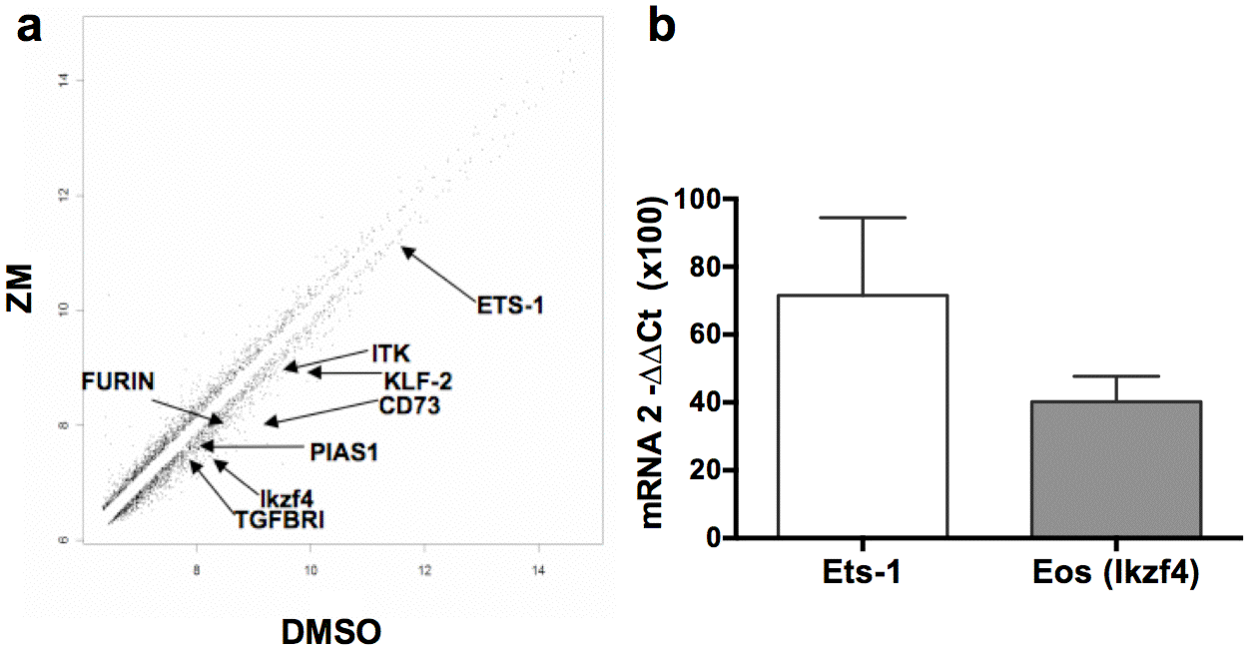
Molecular impact of JAK/STAT signaling inhibition in Treg. **(A)** Scatter plot comparison of average expression values in ZM-treated cells (ZM) versus vehicle-treated cells (DMSO) for all the 45,282 probes in expanded CD4^+^GFP^+^ T cells from Foxp3-eGFP mice treated for 2h in presence of IL-2. **(B)** mRNA quantification by Taqman RT-PCR on Ets-1 and Eos (Ikzf4) in 2hrs ZM-treated cells relative to control values in untreated cells.

In order to determine whether inhibiting JAK/STAT signaling pathway in Treg might impact other genes than Foxp3 in the short term, we performed a comparative analysis of the transcriptome in ZM-treated or untreated CD4^+^GFP^+^ Treg. Using a 1.5-differential fold-change expression cut-off, 110 genes were down-regulated and 51 genes were up-regulated after a 2 hour treatment, including some genes involved in canonical pathways independent or associated with JAK/STAT signaling (*Pias-1*, *Ets-1*) (Fig 5a). Among the 110 down-regulated genes, several have been associated with Treg function and/or regulation of Foxp3 expression, like CD73 [27], furin [28] pias-1 [29], Eos (ikzf4) [30] or Ets-1 [10]. We confirmed by qPCR that Eos mRNA levels were strongly reduced in this experiment, and observed a tendency for the loss of Ets-1 mRNA as well (Fig 5b). The treatment with the ZM inhibitor also altered the expression of a few genes from the Treg signature and of many target genes for Foxp3 (S2 Fig). Of note is that despite the reported affinity for JAK3, genes from signaling pathways other than JAK/STAT were affected by the ZM inhibitor (S2 Fig). Among those, the MAPK pathway might have played a specific role since ERK signaling has been associated with lower expression of Foxp3 [31]. Thus, our results reveal that several genes involved in Treg identity were also maintained by the JAK/STAT pathway in the short term.

## Discussion

To our knowledge, few studies have investigated the impact of JAK/STAT inhibitors on established Treg *in vitro* [32,33] and *in vivo* [34] and never on the very short time scale that we report here. Moreover, dead cells were not excluded from most of these analysis and no molecular data on mRNA levels or methylation of the CNS2 were provided. Thus, our results significantly expand our knowledge on the regulation of Foxp3, a crucial transcription factor for maintenance of immune homeostasis [35,36], and provide clues on how an established Treg may convert to an effector T cells.

Our analysis of the early events after inhibition of the JAK/STAT signaling pathway reveals an unexpected rapid turn over of Foxp3, perhaps the most striking result of our study. We confirmed this rapid turn over using CHX, a wide inhibitor of mRNA translation. Previous studies assessing the stability of Foxp3 expression upon CHX treatment gave values for Foxp3 ‘half-life’ ranging from 20 min to several hours depending on the model [37,38]. Based on the slopes that we obtain with the CHX inhibitor, we would estimate a much shorter half-life for Foxp3 of 9.8 min in murine primary Treg. The similarity in Foxp3 reduction kinetics between JAK3 inhibitors and CHX suggests that most of the turn-over of Foxp3 would depend on JAK3 activity. Furthermore, our qPCR data reveals that the loss of Foxp3 mRNA mirrors the loss of the Foxp3 protein. Altogether, our results suggest a constant input of Foxp3 mRNA at steady state in Treg, leading to the renewal of 50% of new Foxp3 proteins every 10 min or so. In the absence (or strong reduction) of IL-2 signaling, reduced input of mRNA would rapidly translate into the loss of the protein. Our results are in line with the proposition that cellular protein levels are mostly constrained at the mRNA translation level. Moreover, they also concurs with molecular studies showing that transcription factors are in the group of molecules with the shortest half-lives [39]. Further studies are needed to decipher the exact mechanisms behind the control of Foxp3 mRNA by the JAK/STAT signaling pathway, but microRNA might be involved since it has been shown that miR31 directly interact with Foxp3 mRNA to repress expression of the protein [40]. Interestingly, at least one miR (miR182) has been described to be under the control of IL-2 in T cells [41].

Recently, it was demonstrated that the proteasome plays a central role in Foxp3 stability [24,25]. Our results failed to show any significant prevention on the loss of Foxp3 with proteasome inhibitors. However, the USP7 gene, proposed as an important stabilizer of Foxp3 [24], was reduced to a similar extent than Foxp3 following JAK/STAT inhibition, suggesting that a reduction in USP7 activity might have been responsible for directing Foxp3 to an alternative pathway of ubiquitin-dependent degradation. These contrasting results might in part be explained by toxicity of proteasome inhibitors relative to JAK/STAT inhibitors. The exact mechanism by which the Foxp3 protein is lost upon JAK/STAT inhibition remains to be determined.

Supporting the hypothesis that IL-2 signaling is mainly involved in the control of Foxp3 mRNA transcription, we show here that STAT-5 is bound to the GAS-region of the CNS2 upon IL-2 signaling, and released upon inhibition of this signaling. Thus, it is tempting to speculate that a continuous IL-2 signal would be needed for STAT-5 binding to the Foxp3 and subsequent Foxp3 mRNA transcription. This hypothesis is supported by results showing that the CNS2 needs to be 'activated' by IL-2 to maintain Foxp3 expression [13]. However, we report here that the loss of Foxp3 expression was not accompanied by remethylation of the CNS2, showing that Foxp3 expression and methylation of CNS2 are not always associated. This result confirms previous studies performed with murine Treg *in vivo* [20] and with human Treg *ex vivo* [42]. Although these studies showed that IL-2 signaling pathway was defective in human 'exTreg' [42] and that IL-2/IL-2R complexes were able to prevent Treg conversion in vivo [20], our data are the first, to our knowledge, to show that the loss of Foxp3 upon JAK/STAT inhibition is reversible if physiological levels of IL-2 are provided. This strongly suggest that IL-2 is permanently controlling Foxp3 transcription *in vivo*, explaining why pSTAT5 is associated with Foxp3 expression in freshly isolated Treg [22] and in close vicinity with IL-2-producing cells *in vivo* [43].

It has also been reported that the TCR signal is important to maintain Foxp3 due to an adequate chromatin 3D architecture [14]. We found here that the extent of FoxP3 reduction upon JAK/STAT inhibition was similar in TCR-stimulated cells or not. This suggest that the JAK/STAT pathway might be dominant over the TCR pathway to maintain Foxp3 in Treg. In activated or resting Treg, 30 to 40% of Treg 'resisted' the treatment, establishing the proportion of 'plastic' Treg to 60 to 70% of all Treg, a relatively similar figure than the one determined on TCR-activated Treg [13] and slightly higher than the one proposed *in vivo* during EAE [20]. It has been suggested that TGF-ß might play an important role on methylation of CNS2 through STAT-5 [44]. Whether TGF-ß plays a role in remethylation of the CNS2 in the absence of IL-2 signaling remains to be investigated.

In summary, our results significantly expand our knowledge on the regulation of Foxp3 at the cellular and molecular level. We show that Foxp3 has a short half life controlled by the JAK/STAT signaling pathway mostly at the level of mRNA transcription and/or stability. They also establish that loss of Foxp3 can be observed in a context of a demethylated CNS2. 'ExTreg', the existence of which has been disputed in the past [45], have now been described in numerous studies and have their CNS2 remethylated in some [13] but not all [20,42] studies. Thus, we would like to suggest that the population of Foxp3^lo^CD25^+^ cells with a demethylated CNS2 that are generated *in vitro* very rapidly after cessation of the JAK/STAT signaling pathway might represent the precursors of 'exTreg'. These precursors might be rapidly generated *in vivo* in conditions of limited IL-2 availability and/or prolonged inflammatory conditions. A complete understanding on the extra and intracellular signals leading to full dedifferentiation of Treg will be important for a better comprehension of immune homeostasis.

## Materials and Methods

### Mice

C57BL/6J Ly5.2 (CD45.2^+^ CD90.2^+^) mice were purchased from Janvier Labs (Le Genest Saint Isle, France). Foxp3-eGFP C57BL/6 Ly5.2 mice were obtained courtesy of Dr. Bernard Malissen (Centre d'Immunologie de Marseille-Luminy, France). For some experiments, Foxp3-eGFP mice were crossed with congenic CD90.1^+^ mice. All animals were kept under specific pathogen-free conditions and manipulated according to European council directive 86/609/EEC. Briefly, mice were given 3% fat food and acidified water *ad libitum* and were maintained under a 14-hour light 10-hour dark cycle. Animals were euthanized by cervical dislocation after isoflurane anesthesia. The study was approved by the Ethical Committee of Région Ile-de-France (ref p3/2008/039).

### Cell purification and sorting

Human cells were obtained from leuko-apheresis samples freshly collected from healthy donors from the EFS (Etablissement Français du Sang), after informed consent and under an institutional agreement with the EFS. Peripheral blood mononuclear cells (PBMC) were separated on Ficoll-Hypaque gradient (PAA laboratories, Pasching, Austria). CD25^+^ cells were enriched from PBMC using CD25 microbeads II, LS columns and a MidiMACS separator (Miltenyi).

Murine CD25-enrichment was performed on pooled spleens and peripheral lymph nodes cells prepared by mechanical dissociation in PBS 1X supplemented with 3% FCS. Cells were sequentially incubated for 20 minutes at 4°C with saturating amounts of biotin-labeled anti-CD25 mAb (clone 7D4; BD) and then, anti-biotin microbeads (Miltenyi), followed by two rounds of magnetic cell separation in LS columns. Alternatively, cell sorting was performed using the auto-MACS (Miltenyi). A purity of 50% to 60% of CD4^+^CD25^+^ cells, containing 70% to 80% of Foxp3^+^ cells, was regularly observed. Cell sorting from Foxp3-eGFP mice was performed with one LS column, followed by staining with anti-CD4 mAb and sorted with a FACSAria (BD) on the basis of CD4 and GFP expression, ensuring purity greater than 96%.

### In vitro treatments

In most experiments, CD4^+^GFP^+^ purified T cells were cultivated for four to twenty-five days in RPMI 1640 (Life Technologies, Paris, France) supplemented with 10% FCS, 2 mM glutamine, 100 U/ml penicillin, 100 mg/ml streptomycin, 50 μM β-Mercaptoethanol and 10 mM HEPES, with Dynabeads Mouse T-activator CD3/CD28 (Life Technologies, 2 beads for 1 T cell) and 10 ng/ml mIL-2 (Peprotech, Paris, France), at a concentration of 10^6^ cells/ml, and incubated in a 37°C, 5%CO_2_ incubator. Fresh mIL-2 was added every 2–3 days. Cycloheximide (CHX), ZM39923 (ZM) and Tyrphostin/AG490 (AG) (Sigma Aldrich) were diluted in 100% DMSO (CHX, ZM) or absolute ethanol (AG) and were used at final concentrations of 30 μM for ZM, 50–200μg/ml for AG and 10 μg/ml for CHX. The proteasome inhibitors Epoxomycine and MG-132 (Sigma) were diluted in 100% DMSO and were used at a final concentration of 5μM. For experiments with expanded CD4^+^GFP^+^ T cells, inhibitors were added directly in culture medium or 24h after removal of CD3/CD28 Dynabeads. Treatment was performed on 0.1x10^5^ to 10^6^ cells per well at a concentration of 10^6^ cells/ml. For freshly purified or enriched T cells, cells were grown in complete RPMI 1640 medium, in presence of mIL-2 at 10 ng/ml. For human studies, cells were cultivated in RPMI 1640 medium supplemented with 10% human serum, 2% penicillin/streptomycin and 1% L-Glutamine, and with recombinant human IL-2 (600 IU/ml).

To generate TGF-ß-induced Treg, CD4^+^CD25^-^CD44^low^GFP^-^ T cells were purified from spleen and peripheral lymph nodes of Foxp3-GFP mice by flow cytometry cell sorting. Cells were grown for 4 days culture in a 37°C, 5% CO2 incubator with plate-bound anti-CD3 (clone 145-2C11, BD) (1 μg/ml) and anti-CD28 (1 μg/ml), in combination with human TGF-β (Peprotech) (10 ng/ml) and mIL-2 (Peprotech) (10 ng/ml). Anti-CD122 mAb (TMβ1) was prepared by Agro-Bio (La Ferté Saint-Aubin, France) from ascites or obtained from BioXCell (West Lebanon, NH, USA) and was used at a final concentration of 10 μg/ml.

### Flow cytometry and monoclonal antibodies

The following monoclonal antibodies were used for phenotypic analysis: for mice studies: Alexa Fluor 700- or phycoerithrin (PE)-labeled anti-CD4 (clone RM4-5, BD), peridinin-chlorophyll-protein (PercP)-labeled anti-CD8 (clone 53–6.7, BD), PE-Cyanin7 (PECy7)-conjugated, allophycocyanin (APC)- or PE-labeled anti-CD25 (clone PC61, respectively from eBioscience and BD), Pacific blue- or eFluor 450-labeled anti-Foxp3 (clone FJK16s, eBioscience), PE-labeled anti-Foxp3 (clone FJK16s, BD), Alexa Fluor 647-labeled anti-Foxp3 (clone MF23, BD), PercP-labeled CD90.1 (OX-7, BD) and PE-labeled anti-Bcl-2 (clone 3F11, BD). For human studies: PE-labeled anti-CD25 (clone M-A251, BD or 4E3, Miltenyi Biotec, Paris, France), PercP-labeled anti-CD4 (clone SK3, BD), PE-Cyanin7-labeled anti-CD3 (clone UCHT1, Biolegend, San Diego, CA, USA), Alexa Fluor 488- or APC-labeled anti-FOXP3 (clone 236A/E7, eBioscience) and Alexa Fluor 700-labeled anti-CD8 (clone HIT8a, Biolegend). Alexa fluor 647-labeled anti-pSTAT5 (Y694, BD) was used in both mice and human studies.

For cell surface staining, 10^5^ to 10^6^ cells were labeled with 50–100 μl of a mix of mAbs at proper concentrations in PBS containing 3% FCS for 20–30 minutes at 6–8°C in the dark. Staining with the Fixable Viability Dye eFluor 780 (eBioscience, Paris, France) or with the Live Dead viability assay Green dead cell stain kit (Life Technologies, Saint Aubin, France) was performed before cell surface staining, according to the manufacturer’s instructions. Intracellular staining using Foxp3 mAb was performed according to the manufacturer’s instructions (eBioscience). Detection of pSTAT5 was already described in details [22]. Briefly, cell suspensions were fixed with 10 volumes of PBS 1X 1.5% formaldehyde for 10 minutes at room temperature. Cells were then washed in PBS 1X 0.2% BSA, and permeabilized with 100% methanol for 10 minutes on ice. After extensive washing, cells were incubated for 45 minutes in the dark at 6–8°C. Fluorescent signals were collected on a FACS LSR II (Becton Dickinson (BD), San Diego, CA, USA). Proper compensation using Fluorescence Minus One (FMO) controls were established with the FlowJo software (TreeStar, Ashland, OR). To accommodate for variations in the MFI of Foxp3 and CD25 among different experiments, results are presented as percentages of controls, representing an index between the MFI of the molecule of interest in presence of the inhibitors divided by the MFI of the same molecule in presence of the vehicle control. In some experiments, the delta between the MFI of Foxp3^+^ and Foxp3^-^ cells in the same sample was also taken into account for the calculations.

### Foxp3, USP7 and STUB1 RT-PCR assay

Reverse transcription was performed on 10^6^ AG- or EtOH-treated CD4^+^GFP^+^ expanded Treg cells from Foxp3-eGFP reporter mice. RNA was extracted from cell pellets using the RNAeasy extraction kit (Qiagen, Hilden, Germany). Then, reverse transcription was performed with the kit Superscript III (Life Technologies). Finally, cDNA was amplified by real-time PCR with the Taqman Gene expression assay (Applied Biosystems-Life Technologies) following the manufacturer’s instruction. Real-time PCR was performed with a ABI PRISM^®^7700 Sequence Detector (Applied Biosystems, Carlsbad, CA, USA). Quantitation of mRNA expression of Foxp3 in AG-treated vs EtOH-treated cultures was determined using the 2^-ΔΔC^_T_ hypothesis according to the following formula: 2^-[(C^_T_^Foxp3-C^_T_^GAPDH) AG- (C^_T_^Foxp3-C^_T_^GAPDH) EtOH]^

### Western Blot

Proteins were extracted from cell pellets in lysis buffer (RIPA, Thermo Scientific) containing protease inhibitor cocktail (Pierce) and phosphatase inhibitor cocktail (Sigma). Proteins were separated in precast NuPAGE Novex 4–12% Bis Tris Protein Gels in NuPage MOPS SDS running buffer and transferred to PVDF membranes with iBlot system (all from Life Technologies). After incubation with anti-Foxp3 (Cell Signaling) or anti-beta Actin (Abcam) overnight at 4°C, proteins were detected using species-specific biotinylated secondary antibodies and Qdot 625 streptavidin conjugate (Invitrogen). Signals were quantified by densitometry with ImageJ 1.45 software (NIH, Bethesda, USA).

### Chromatin immunoprecipitation and methylation assay

ChIP assay was performed using the Magnify ChIP system, according to the manufacturer instructions (Life Technologies). Briefly, cells in culture were fixed using formaldehyde-based buffers. Chromatin was sheared by brief pulse of ultrasound on ice and anti-STAT5 (clone ST5b-10G1, mouse anti-human STAT5b, Life Technologies) or control mouse IgG (Life Technologies) was used to pull-out the transcription factor associated to the chromatin. Fixation was reverted and DNA was purified using magnetic beads. This DNA material was subjected to quantitative PCR using SYBR Green Master mix (Life Technologies) on a ABI PRISM^®^ 7700 Real Time PCR System. Primers used were specific for the putative STAT5 binding site in the TSDR region of the murine *foxp3* gene: foxp3-for (5’→3’): AGAACTTGGGTTTTGCATGG;foxp3-rev (5’→3’): ACTTGGCCAGATTTTTCTGC. Relative occupancy was calculated according to the following formula: 2 ^(Ct^_NegCtrl_−^Ct^_Target_^)^ x100 where CtNegCtrl and CtTarget are median threshold cycles of PCR done in triplicates on DNA samples from the Negative Control ChIP (using non-immune IgG) and targeted ChIP (using specific antibody) respectively.

Genomic DNA was isolated from ZM- or DMSO-treated CD4^+^GFP^+^ expanded Treg cells of Foxp3-eGFP mice using the DNeasy tissue kit (Qiagen) following the supplier's recommendations. Bisulphite sequencing was performed as described [46].

### DNA microarray hybridization and analysis

RNA from 2x10^6^ DMSO- or ZM39923-treated expanded CD4^+^GFP^+^ cells of Foxp3-eGFP mice was generated using RNeasy Mini kit (QIAGEN) and its quality was verified in an Agilent Bioanalyzer. Total RNA was amplified and converted to biotinylated cRNA according to the manufacturer’s protocol (Illumina TotalPrep RNA Amplification Kit; Ambion). Two biological duplicates were hybridized to the Sentrix BeadChips Array mouse WG-6 v2 (Illumina). Heatmap representation of log2 expression data was generated using R and Treeview software using the Euclidean correlation similarity measure and complete linkage algorithm. Scatter plot was performed using a log2 representation.

### Statistical analyses

Two-tailed unpaired t test with 95% confidence intervals were performed using GraphPad Prism version 5.0 for PC (GraphPad Software, San Diego, CA). Mean values were considered statistically different if the p value was below 0.05.

## Supporting Information

S1 Fig Reduction of Foxp3 upon JAK/STAT inhibition is not compensated by TGF-ß signaling. (PDF)

S2 Fig JAK inhibitors affect several genes involved in Treg identity. (PDF)

## Abbreviations

Treg: regulatory T cells
TSDR: Treg-Specific Demethylated Region
CNS2: Conserved Non Coding Sequence 2
CHX: cycloheximide
ZM: ZM-39923
AG: AG-490 (Tyrphostin)
