# Correction: Inhibition of the JAK/STAT Signaling Pathway in Regulatory T Cells Reveals a Very Dynamic Regulation of Foxp3 Expression

**DOI:** 10.1371/journal.pone.0157629

**Published:** 2016-06-09

**Authors:** The PLOS ONE Journal Staff

There are errors in the Funding section. The correct funding information is as follows:

This study was funded by grants from the ANR (Agence Nationale de la Recherche to BLS and GM), the AFM (Association Francaise contre les Myopathies to GM), the ANRS (Agence Nationale pour la Recherche contre le SIDA to GM) and by the German Research Foundation (CRC738 and CRC854 to JH). The funders had no role in study design, data collection and analysis, decision to publish, or preparation of the manuscript.

Additionally, there are errors in the author initials for the Author Contributions section. The correct contributions are as follows: Conceived and designed the experiments: JDG GM. Performed the experiments: JDG AB BZ KS JKP GM. Analyzed the data: JDG AB BZ KS JKP JH EP BLS GM. Contributed reagents/materials/analysis tools: JKP JH EP BLS. Wrote the manuscript: JDG JH EP BLS GM.

Additionally, there is an error in reference 2. The correct reference is: Bennett CL, Christie J, Ramsdell F, Brunkow ME, Ferguson PJ, Whitesell L, et al. The immune dysregulation, polyendocrinopathy, enteropathy, X-linked syndrome (IPEX) is caused by mutations of FOXP3. Nat Genet. 2001 Jan 1;27(1):20–1.

Finally, there is an error in the labeling of panel B in Fig 1. Please view the corrected Fig 1 here.

**Fig 1:**
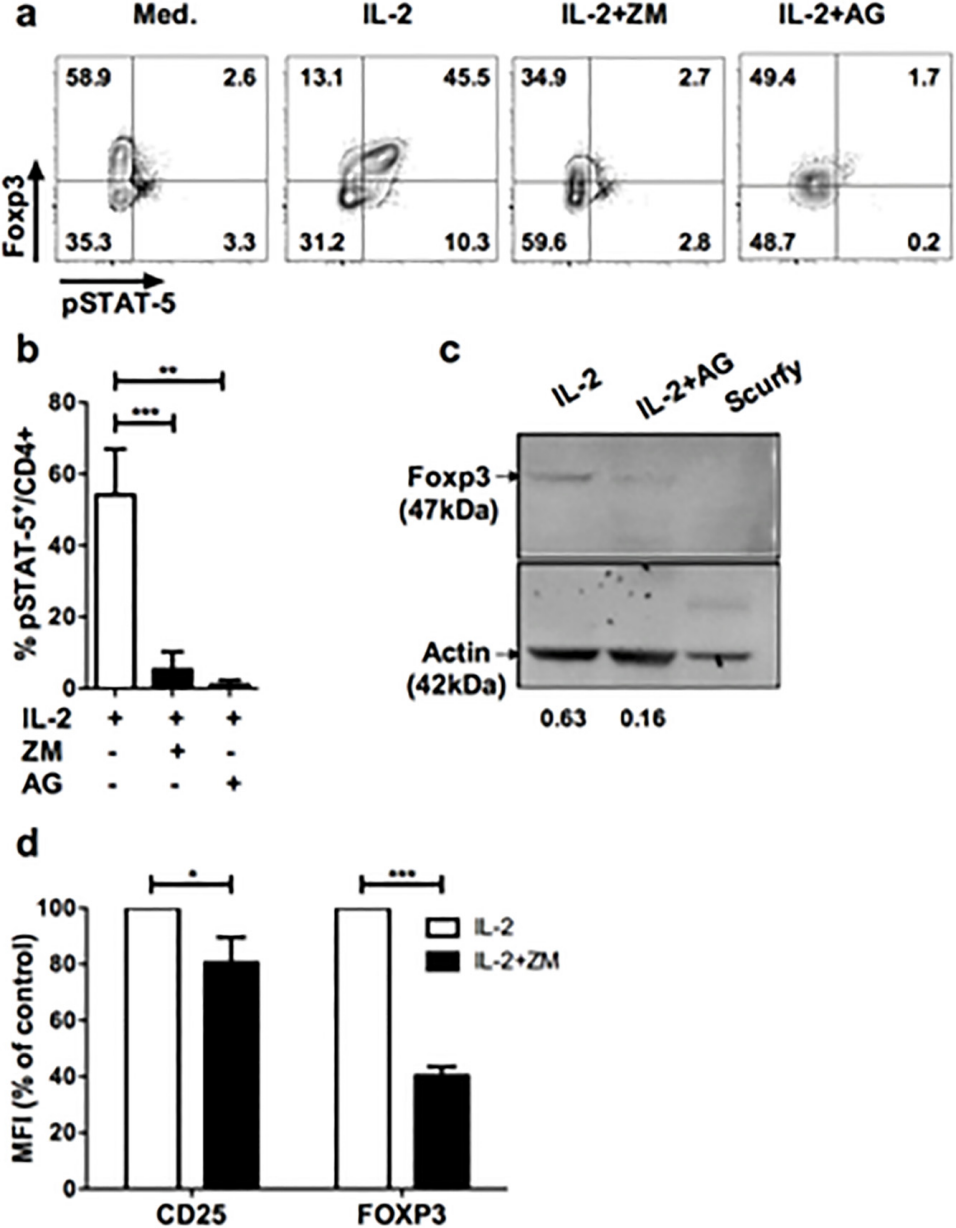
Blockade of JAK/STAT signaling pathway leads to down modulation of Foxp3 in Treg. (**a**) CD25-enriched T cells were cultured for one hour in complete medium alone (Med.), with IL-2 (IL-2), or IL-2 supplemented with ZM-39923 (IL-2+ZM) or AG-490 (IL-2+AG). Profiles shown are gated in CD4^+^ cells and are representative of 4 independent experiments. (**b**) Frequencies of pSTAT5^+^ cells among CD4^+^ cells cultured for one hour with IL-2 alone (IL-2) or in presence of IL-2 and the indicated JAK inhibitors (ZM, AG). Results are compiled from 4 independent experiments. **(c)** In vitro expanded CD4^+^GFP^+^Treg were treated with IL-2 and with either 100ug/mL of AG490 (IL-2+AG) or vehicle control (absolute ethanol; EtOH) for 2 hrs (IL-2). Proteins were extracted in lysis buffer and blotted followed by anti-Foxp3 and anti-actin staining. Intensity values were normalized to the actin band in each blot. The specificity of the Foxp3 staining is attested by the absence of the Foxp3 band in extracts from splenocytes of a scurfy (genetically deficient for Foxp3) mouse. **(d)** Human CD4^+^CD25^+^ cells were enriched from PBMC of healthy donors by magnetic sorting and treated with IL-2 (600 IU/mL) with or without ZM (50 μM) for 1h. MFI of CD25 and FOXP3 in human CD4^+^CD3^+^ cells after treatment with IL-2 alone (IL-2) or IL-2 in presence of ZM (IL-2+ZM). The MFI of Foxp3 and CD25 shown were normalized by the MFI of control cultures. These data are compiled from 3 independent experiments. Statistical significance was tested using Student t-test (***p<0.001, **p<0.01, *p<0.05).
