# Supplementary material for: Inhibition of the JAK/STAT Signaling Pathway in Regulatory T Cells Reveals a Very Dynamic Regulation of Foxp3 Expression
Source: PLoS One. 2016 Apr 14;11(4):e0153682. doi: 10.1371/journal.pone.0153682 (PMC4831811; doi:10.1371/journal.pone.0153682)
Supplement: S1 Fig — (PDF) [file pone.0153682.s001.pdf]

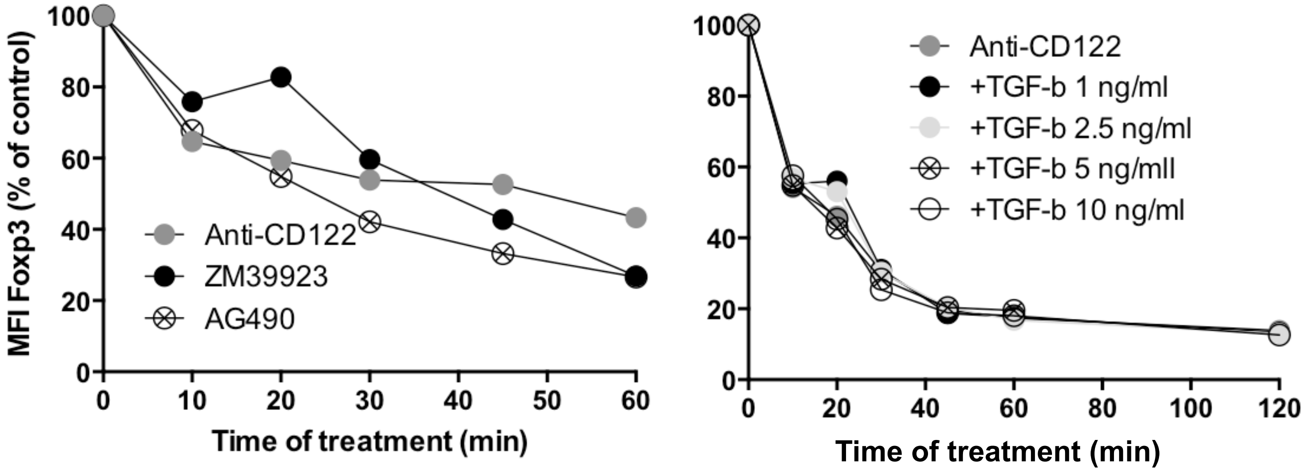

S1 Figure. Reduction of Foxp3 upon JAK/STAT inhibition is not compensated by TGF-ß signaling. (A) Median fluorescence intensity (MFI) of Foxp3 in TGF-b-induced Treg relative to untreated cells in live $CD4^+$ cells upon the treatments indicated in the legend. These data originate from a single experiment. **(B)** MFI of Foxp3 in sorted Foxp3-GFP Treg treated with the indicated dose of TGF-b and anti-CD122 mAb. Numbers represent the ratio of between the MFI determined in the test relative to the MFI of Foxp3 in untreated cultures. These data are representative of one experiment out of two**.**
