# Supplementary material for: Inhibition of the JAK/STAT Signaling Pathway in Regulatory T Cells Reveals a Very Dynamic Regulation of Foxp3 Expression
Source: PLoS One. 2016 Apr 14;11(4):e0153682. doi: 10.1371/journal.pone.0153682 (PMC4831811; doi:10.1371/journal.pone.0153682)
Supplement: S2 Fig — (PDF) [file pone.0153682.s002.pdf]

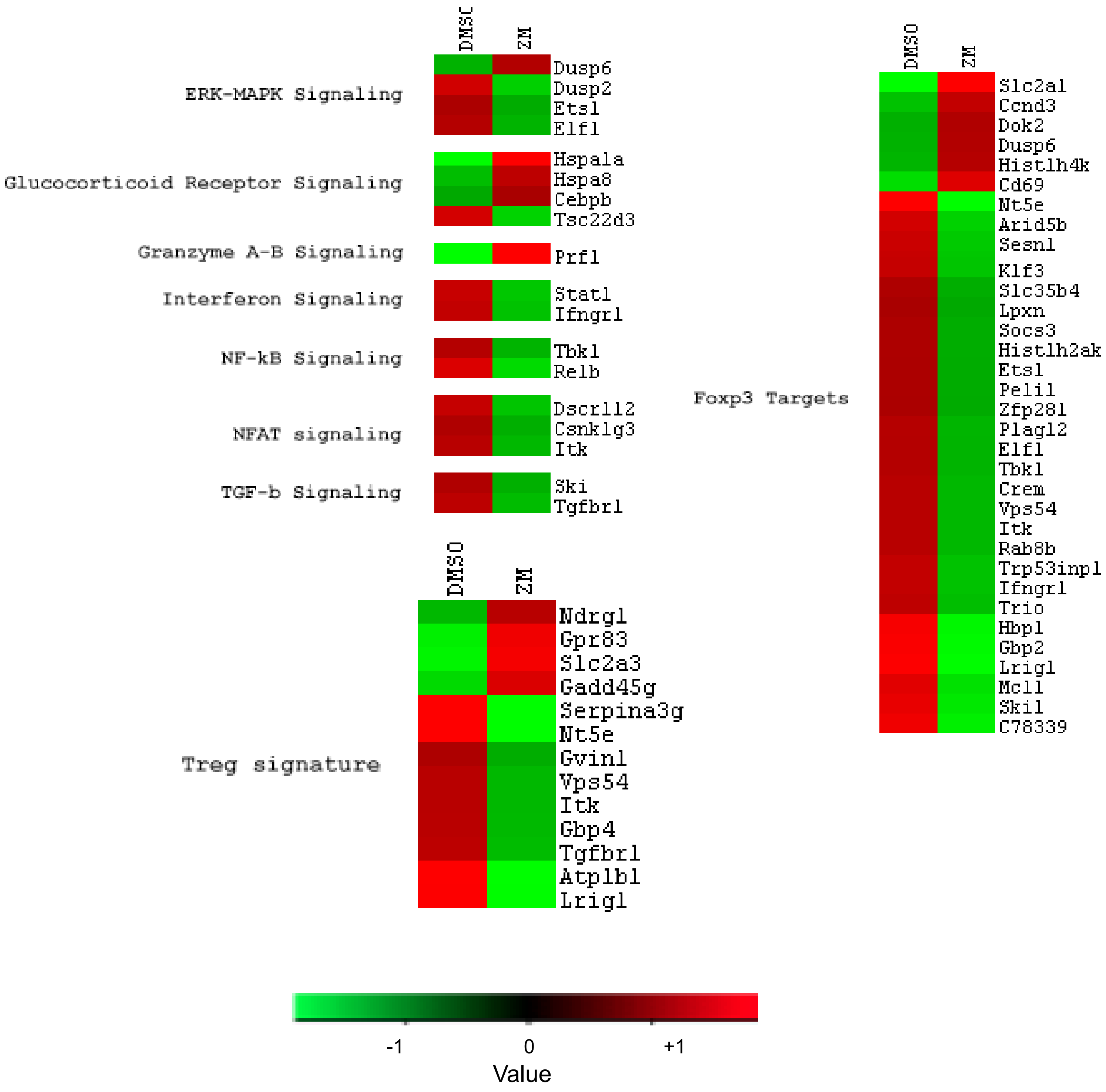

S2 Figure. JAK inhibitors affect several genes involved in Treg identity. Modulation of the indicated genes after treatment of Treg with the JAK3 inhibitor ZM39923 (ZM) or vehicle control (DMSO) for 2 hrs are listed according to their implication in the indicated signaling pathways or if they belong to the Treg signature or Foxp3 targets. Only genes with changes of 1.5 fold or more are shown.
